# Interpretable machine learning for yak milk feeding pattern discrimination: Integrating XGBoost with multidimensional explainability analysis

**DOI:** 10.1016/j.fochx.2026.103541

**Published:** 2026-01-14

**Authors:** Bo Hu, Lu Sun, Haiyue Wu, Rong Hu, Zhongxin Yan

**Affiliations:** Qinghai Academy of Animal Science and Veterinary Medicine, Qinghai University, Xining 810016, China

**Keywords:** Yak milk, Machine learning, Extreme gradient boosting, SHAP, Grazing authentication, Model interpretability

## Abstract

Accurately identifying grazing (GZ) and supplementary feeding (SF) patterns in yak milk is important for product authentication; however, current methodologies are often expensive and time consuming. In this study, we examined 523 milk samples of lactating yaks at four stages of SF and tested 21 machine learning algorithms to develop a rapid and cost-effective classification method using routine compositional parameters. Ensemble learning techniques performed better than others, with XGBoost having the best accuracy (92%) and AUC (0.94). Multidimensional interpretability analyses, including SHAP, PDP, and ICE, identified fat content (27.8%) and lactose (23.1%) as the most important discriminators, along with biologically meaningful interactions, such as between fat, lactose, and freezing point. This interpretable framework provides a practical, low-cost method for milk authentication of yaks using ordinary dairy analyzers, providing a methodological foundation for the establishment of standardized GZ certification systems in milk production from yaks.

## Highlights

• XGBoost achieved 92% accuracy for high-precision yak milk feeding pattern detection.
• SHAP, PDP, and ICE methods explained XGBoost model decision mechanisms.
• Routine milk composition data enables cost-effective feeding pattern classification.
• SHAP revealed fat-freezing point and lactose-freezing point interaction effects.

## Graphical abstract

**Figure:**
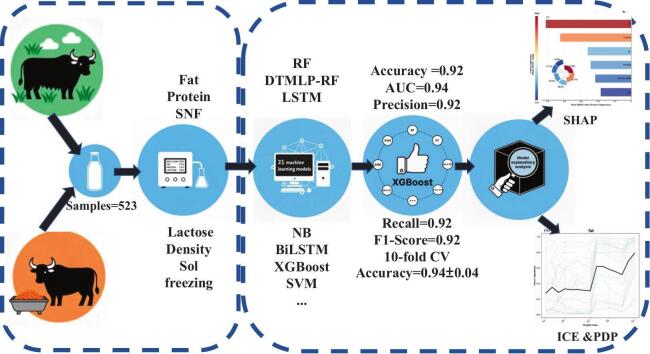

## 1 Introduction

The yak (*Bos grunniens*) is widely distributed over the highlands at altitudes of 3000 to 5000 m. Its milk is said to be a natural concentrate, being rich in protein and other nutrients (Li et al., 2023). In recent years there has been a greater willingness to pay a premium for milk from exclusively grazed cattle by consumers (Wang et al., 2025). However, in comparison to conventional bovine milk, the authentication of grazing (GZ) vs. supplementary feeding (SF) yak products is difficult because of harsh environmental conditions, fragmented production systems and widespread dependence on manual milking (Dong et al., 2003).

Several laboratory techniques have been developed to confirm feeding habits in ruminants. For example, Prache et al. (2020) used FTIR for measuring β-carotene and Soyeurt et al. (2022) used gas chromatography for fatty acids. Although effective, these approaches are expensive, time consuming, and require specialized personnel, limiting their feasibility for large-scale field application. Rapid milk composition analyzers are a low-cost alternative (Yang et al., 2020); however, conventional analyses are not able to directly discriminate feeding regimes. Machine learning (ML) addresses this shortcoming by automatically identifying latent patterns in high dimensional data sets and has been successfully implemented for milk source identification, quality assessment (Frizzarin et al., 2021; Wang, Zhang, et al., 2023), and safety monitoring (Guo et al., 2025; Sadeghi Vasafi & Hitzmann, 2022). Nonetheless, previous studies have mostly been conducted on traditional dairy breeds such as Holstein with little research on yak milk. Moreover, most ML models are “black boxes” that lack the transparency needed to be used in food certification systems. Considering the limitations of single explanation techniques (Arenas et al., 2021), we used a multidimensional interpretability strategy combining SHAP (Lundberg & Lee, 2017), partial dependence plots (PDP) and individual conditional expectation (ICE) to understand the underlying decision-making processes.

Since protein variation has been shown to best reflect the SF intensity (Miller, 2004) on the Qinghai-Tibet Plateau, we collected 523 milk samples under controlled GZ and SF regimes to systematically evaluate 21 ML algorithms. Building on this evaluation, we created a high-precision discrimination framework, which combines ML with multidimensional interpretability analyses. This approach allows for rapid and economical authentication based on routine milk composition data and lays the basis for a methodology for quality control in the yak dairy industry.

## 2 Materials and methods

### 2.1 Statement of ethics

This study was approved by the Animal Ethics Committee of Qinghai Academy of Animal Husbandry and Veterinary Science (No. 2024-QHMKY-006). All procedures were conducted in accordance with the Chinese standard GB/T 35892–2018 (Guidelines for Ethical Review of Welfare of Experimental Animals) and the NIH (National Research Council) Guide for the Care and Use of Laboratory Animals (8th ed., 2011).

### 2.2 Experimental design and animal management

#### 2.2.1 Experimental sites

The experiment was carried out at the same time at two sites in Qinghai Province from 20 July to 16 November 2023, covering 110 days, including a 10-day adaptation period. The Tianjun County site (99° 10′40.56″E, 37° 24′53.79”N) is located at an altitude of 3700 m and the Gonghe County site (100° 49′15.23″E, 36° 17′42.68”N) is located at an altitude of 2900 m. Both locations have a typical continental plateau climate and are the typical ecological conditions of major areas for yaks production on the Qinghai-Tibet Plateau.

#### 2.2.2 Experimental animals and group design

At each site, 70 healthy multiparous lactating female yaks (aged 4–6 years) of similar body weights and lactation stages were selected, all of which were managed under traditional extensive GZ. The yaks were randomly allocated to four treatment groups, a control group (GZ, *n* = 56, GZ only), and three different protein levels of SF (low (LP), medium (MP), and high (HP), *n* = 28 in each group).

The experiment lasted 110 days (20 July to 16 November 2023), with a 10-day adaptation period and was divided into four 30-day periods (D_1_-D_4_, August to November). Yaks in the SF groups were supplemented with 2 kg/day of concentrate feed from 08:00 to 08:30, based on Qinghai Province standard DB63_T 2042–2022. All animals had free access to water during the study.

For the collection of milk, calves were used to stimulate let down before manual milking. A 100 mL milk sample was taken from each yak and kept at 4 °C and analyzed within 2 h, for a total of 523 samples.

For the purposes of data analysis, the study used a binary classification model by lumping the three SF groups into one category. This approach is a reflection of practical herding variations, is consistent with market certification priorities that are mainly to identify pure GZ milk, and leaves room in the model to capture general characteristics of SF and avoid overfitting to specific protein levels.

#### 2.2.3 Formulation of different protein level feeds

To simulate the practical GZ situation with SF, we formulated three diets with different protein content (Low (LP, 15%), Medium (MP, 17%) and High (HP, 19%) as mentioned in Table 1. While keeping an isoenergetic density (11 MJ/kg ME), the ingredient proportions were adjusted in order to reach these protein gradients: the percentage of protein-rich ingredients (rapeseed and soybean meal) and bran were increased with higher protein contents, whereas the energy sources (corn and wheat) were decreased. This strategy made sure that protein content was the only experimental variable. All diets contained 4% premix and 1% salt to meet the trace elements and mineral needs of the yaks.

**Table 1:** Experimental diet composition and nutritional level table.

Item	LP / %	MP%	HP%
Corn	44	40	37
Wheat	26	18	13
Bran	10	16	17
Rapeseed Meal	10	13	18
Soybean Meal	5	8	10
4% Premix	4	4	4
Salt	1	1	1
Total	100	100	100
ME/(MJ/kg)	11	11	11
CP	15	17	19
EE	3.92	4.29	4.65
CF	4.25	5.15	5.75
NDF	19.51	20.87	21.10
ADF	8.12	9.01	9.63
Ca	0.17	0.19	0.22
P	0.20	0.24	0.28

**Note**: The table provides the ingredient percentages and nutritional analysis for three experimental diets: low protein (LP), medium protein (MP), and high protein (HP). Key metrics include metabolizable energy (ME), crude protein, crude fat (EE), crude fiber, neutral detergent fiber, acid detergent fiber.

### 2.3 Milk sample collection and analysis

To reduce bias, a single-blind design was used: sampling personnel were notified of animal identification numbers only, not treatment assignments. Milk samples (100 mL) were taken from each of the yaks after calves were briefly separated and allowed to suckle to stimulate letdown prior to milking by hand. Samples were kept at 4 °C and analyzed in a period of 2 h with a Julie Z10 analyzer (Fulmatic™, Bulgaria), which was specifically calibrated for yak milk. All measurements were carried out in cow mode at a room temperature of 15–20 °C, giving a total of 523 samples for compositional analysis.

### 2.4 ML methodology

#### 2.4.1 Feature selection

The initial data set included seven continuous variables (fat, protein, non-fat solids, lactose, density, soluble solids, and freezing point) and one categorical variable (the month, D1-D4). Since the months represent qualitative transitions in forage type (from green to dry grass), one-hot encoding was used for the month variable, bringing the total number of features to eleven. Feature selection was then conducted using RF-RFE with 5-fold cross validation and six key features were retained for model construction: fat, protein, non-fat solids, lactose, freezing point and D_4_ (November).

#### 2.4.2 Model construction

To build a baseline of performance, we first tested four classical ML algorithms: k-nearest neighbors (KNN), naive bayes (NB), decision tree (DT) and support vector machine (SVM). In order to better represent the highly nonlinear relationships in the data, we then introduced ensemble learning algorithms, such as random forest (RF), extreme gradient boosting (XGBoost), light gradient boosting machine (LightGBM), categorical boosting (CatBoost), adaptive boosting (AdaBoost) and bootstrap aggregating (Bagging). In addition, three hybrid models, SVM-RF, multi-layer perceptron-RF (MLP-RF), and multi-layer perceptron-DT (MLP-DT) were included. Finally, deep learning methods were used to study even more complex patterns in the dataset.

The deep learning methods were simple neural networks, including multi-layer perceptron (MLP) and convolutional neural network (CNN); sequential models, including long short-term memory (LSTM), gated recurrent unit (GRU), and bidirectional LSTM (BiLSTM); and three deep learning architectures, including CNN-LSTM, CNN-GRU, and CNN-BiLSTM.

#### 2.4.3 Model performance evaluation

We developed a multidimensional evaluation framework to measure the classification performance, discriminative ability and model stability. Accuracy, precision, recall, specificity and F1-score were calculated using the confusion matrix (Eqs. 1–5). Discriminative ability was assessed using the Area under the ROC curve (AUC, Eq. 6), whereas 10-fold stratified cross validation was used to test model stability and generalization performance (Eqs. 7–9).

(1) $$\mathrm{Accuracy}=\frac{\mathrm{TP}+\mathrm{TN}}{\mathrm{TP}+\mathrm{TN}+\mathrm{FP}+\mathrm{FN}}$$

(2) $$\mathrm{Precision}=\frac{\mathrm{TP}}{\mathrm{TP}+\mathrm{FP}}$$

(3) $$\mathrm{Recall}=\frac{\mathrm{TP}}{\mathrm{TP}+\mathrm{FN}}$$

(4) $$\begin{array}{c} \mathrm{Specificity}=\frac{\mathrm{TN}}{\mathrm{TN}+\mathrm{FP}} \end{array}$$

(5) $$\begin{array}{c} F1-\mathrm{Score}=2\times \frac{\mathrm{Precision}\times \mathrm{Recall}}{\mathrm{Precision}+\mathrm{Recall}} \end{array}$$

(6) $$\begin{array}{c} \mathrm{AUC}=\int_{0}^{1}\;\mathrm{TPR}\left(t\right)\cdot \mathrm{dFPR}\left(t\right) \end{array}$$

(7) $$\begin{array}{c} \mu =\left(\frac{1}{K}\right)\sum {ᵢ}^{=1}ᴷ\;Scoreᵢ \end{array}$$

(8) $$\begin{array}{c} \sigma =\sqrt{\left[\left(\frac{1}{K}\right)\sum {ᵢ}^{=1}ᴷ\;{\left(\mathrm{Scoreᵢ}-\mu \right)}^{2}\right]} \end{array}$$

(9) $$\begin{array}{c} \mathrm{CV}=\left(\frac{\sigma }{\mu }\right)\times 100\% \end{array}$$

#### 2.4.4 Explainability analysis methods

We used the game theory-based SHAP method to quantify the contribution of each feature to the model's predictions (Eq. 10). This was combined with PDP to visualize the average marginal effect of each feature (Eq. 11) and ICE plots to capture sample-level heterogeneity (Eq. 12).

(10) $$\begin{array}{c} \phi_{j}=\sum_{S\subseteq F\setminus \left\{j\right\}}\frac{\left|S\right|!\left(\left|F\right|-\left|S\right|-1\right)!}{\left|F\right|!}\left[f_{S\setminus j}\left(x_{S\setminus j}\right)-f_{S}\left(x_{S}\right)\right] \end{array}$$

(11) $$\begin{array}{c} {\mathrm{PDP}}_{j}\left(z\right)=\frac{1}{n}\sum_{i=1}^{n}f\left(x_{1}^{\left(i\right)},\ldots ,x_{j-1}^{\left(i\right)},z,x_{j+1}^{\left(i\right)},\ldots ,x_{p}^{\left(i\right)}\right) \end{array}$$

(12) $$\begin{array}{c} {\mathrm{ICE}}_{j}^{\left(i\right)}\left(z\right)=f\left(x_{1}^{\left(i\right)},\ldots ,x_{j-1}^{\left(i\right)},z,x_{j+1}^{\left(i\right)},\ldots ,x_{p}^{\left(i\right)}\right) \end{array}$$

### 2.5 Statistical analysis and data processing

All the data preprocessing and statistical analyses were performed in Matlab R2023b (MathWorks Inc., Natick, MA, USA), and Python 3.8.10 (Python Software Foundation, Wilmington, DE, USA) was used for the XGBoost model and interpretability analyses. The dataset was randomly split into training (*n* = 417), validation (*n* = 53) and test (n = 53) sets using stratified sampling. All the continuous variables were standardized using *Z*-score normalization using the mean and standard deviation of the training set.

Traditional and deep learning models were implemented with the Statistics and ML Toolbox (v. 12.4) and Deep Learning Toolbox (v. 14.5) of the mathematical software package, Matlab, respectively. The XGBoost model was created with the xgboost package (v. 1.7.5, XGBoost Developers) in Python. For the interpretability of the models, SHAP analyses were conducted using the SHAP package (v. 0.42.1, Scott Lundberg) and the sklearn.inspection module of scikit-learn (v. 1.3.0). All the Python outputs were easily incorporated into the Matlab workflow using the Python interface of Matlab.

## 3 Results

### 3.1 Statistical analysis

**Fig. 1:**
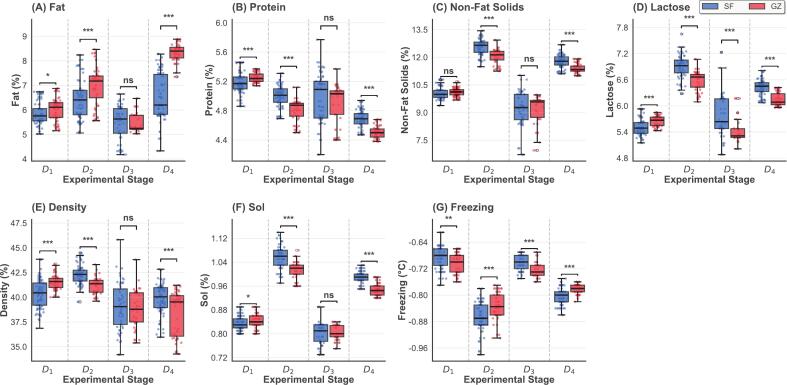
**Temporal variation of milk composition parameters across experimental stages.** Figure caption: Boxplot comparisons of seven milk composition parameters between GZ (red) and SF (blue) groups across four experimental stages (D_1_- D_4_, August–November). Each panel shows: (A) Fat content (%), (B) Protein content (%), (C) Non-fat solids (%), (D) Lactose (%), (E) Density (%), (F) Sol (%), and (G) Freezing point (°C). Box boundaries represent the 25th and 75th percentiles, horizontal lines within boxes indicate medians, and whiskers extend to minimum and maximum values. Statistical significance between groups is indicated by asterisks: *** *p* < 0.001, ** *p* < 0.01, * *p* < 0.05, ns = not significant. Sample sizes: GZ *n* = 225, SF *n* = 298. (For interpretation of the references to colour in this figure legend, the reader is referred to the web version of this article.)

**Fig. 2:**
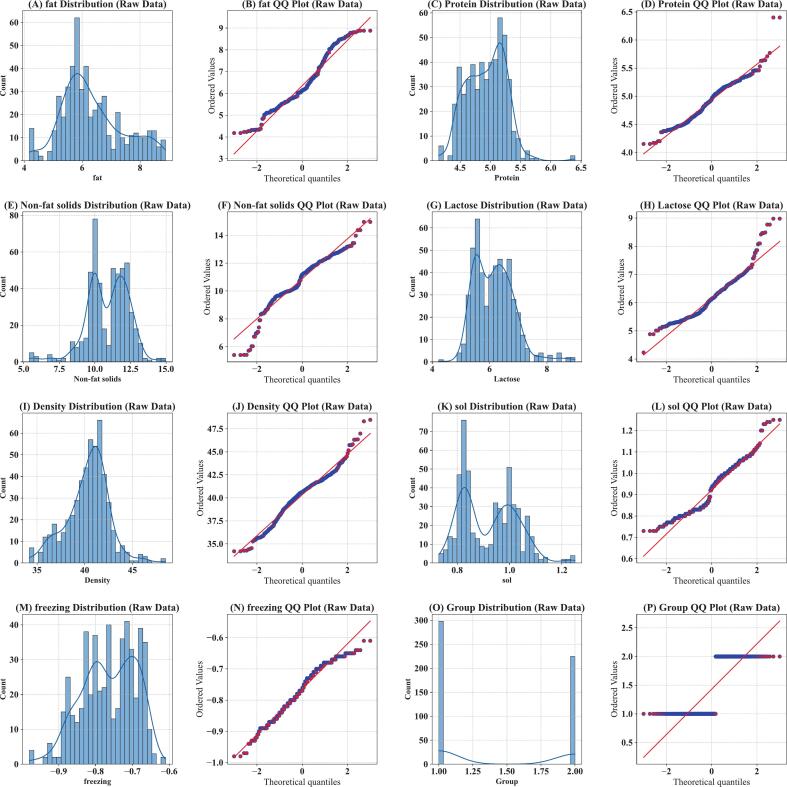
Distribution analysis and normality evaluation of yak milk composition parameters Frequency distributions (left panels) and Q-Q plots (right panels) of 8 parameters from 523 yak milk samples across four feeding groups. Blue bars show sample counts with kernel density curves; blue dots in Q-Q plots represent observed quantiles relative to theoretical normal distribution (red line). (A-B) Fat content, (C—D) Protein content, (*E*-F) Non-fat solids, (G-H) Lactose content, (I-J) Density, (K-L) sol, (M-N) Freezing point, (O—P) Group classification. Lactose and non-fat solids exhibited distinct bimodal distributions, distinguishing SF (Group 1.0, *n* = 298) from GZ systems (Group 2.0, *n* = 225). Most continuous variables demonstrated normal distributions and closely followed theoretical quantiles, validating parametric modeling assumptions. (For interpretation of the references to colour in this figure legend, the reader is referred to the web version of this article.)

Statistical analysis of the composition of yak milk showed that GZ and SF systems had different effects on chemical composition of milk during the experimental period (Fig. 1, Fig. 2). Temporal analysis of the four experimental stages (D_1_-D_4_) showed dynamic compositional changes in response to feeding regimes (Fig. 1). Fat content was significantly greater in GZ in D_1_ (*p* < 0.05), D_2_ and D_4_ (*p* < 0.001) and increased over time, whereas protein showed an opposite decreasing trend. Lactose and freezing point had significant differences at all stages (*p* < 0.05). Interestingly, fat, protein, density, non-fat solids and soluble solids converged at D_3_ with no significant differences, which may reflect the transition from green to withered pasture during this stage.

Distribution analysis (Fig. 2) further showed that non-fat solids, lactose and soluble solids show bimodal distributions (Fig. 2E, G, K), indicating their potential to discriminate between GZ and SF feeding patterns. In contrast, important nutritional components such as fat (Fig. 2A) and protein (Fig. 2C) showed approximately unimodal, normal distributions, with the normality verified by Q-Q plots. The mean values of all components were in accordance with the previously reported ranges for yak milk (Li et al., 2011; Li et al., 2023; Zhu et al., 2021). Together, these temporal and distributional patterns gave a good base of data for ML classification.

### 3.2 Model comparison

**Fig. 3:**
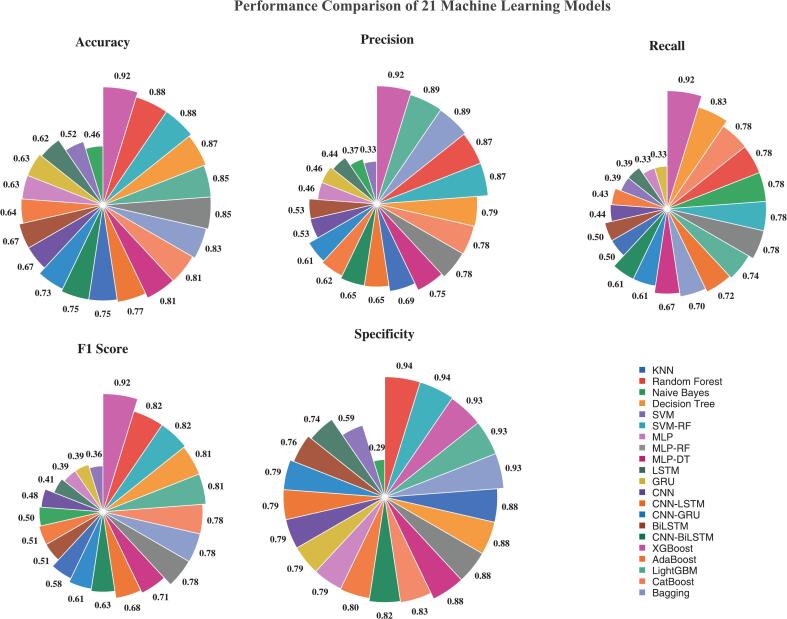
Rose chart showing performance metrics of different classification models This chart provides a visual comparison of the 21 ML models evaluated in the study across five key performance metrics: Accuracy, Precision, Recall, F1 Score, and Specificity. Each colored line represents a different model, as detailed in the chart's legend. This visualization highlights how different models vary in their predictive strengths across the evaluation criteria. (For interpretation of the references to colour in this figure legend, the reader is referred to the web version of this article.)

**Fig. 4:**
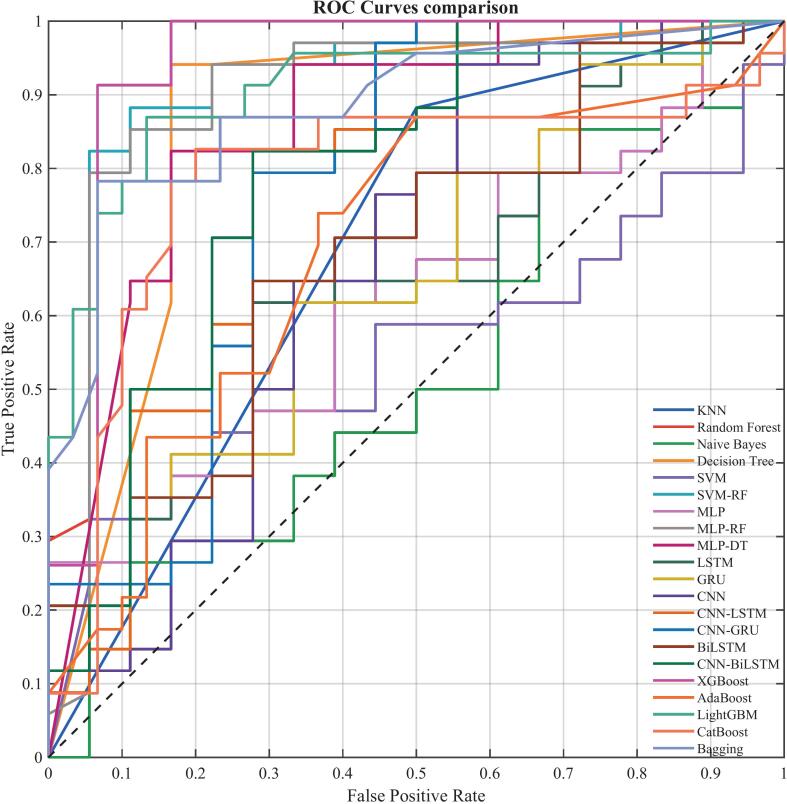
ROC curve comparison of different classification models: showing diagnostic performance of 21 classification models. The x-axis represents False Positive Rate, and the y-axis represents True Positive Rate. The dashed line represents random classifier (AUC = 0.5). ROC curves and their AUC values show different classification performances, with curves closer to the upper left corner indicating better model performance.

We tested 21 ML algorithms for the discrimination of the feeding pattern of the yak milk, as summarized in Table 2 and Fig. 3. XGBoost had the best performance with an accuracy of 92% and a cross-validation accuracy of 94 ± 4%. ROC curve analysis (Fig. 4) further confirmed its superiority, as XGBoost achieved an AUC of 0.94, compared to 0.93 with RF, 0.86 with DT, and 0.78 with CNN-LSTM. RF and SVM-RF also performed well and achieved 88% accuracy. Traditional algorithms showed moderate performance, where DT had 87%, SVM had 52%, and NB has 46%. Deep learning models generally performed worse than ensemble methods.

**Table 2:** Performance Comparison of 21 Machine Learning Models for Yak Milk Feeding Pattern Classification.

ML model	Test							Validation			Train		
	Accuracy	10-fold CV Acc	AUC	F1-Score	Precision	Recall	Specificity	AUC	Accuracy	F1-Score	AUC	Accuracy	F1-Score
XGBoost	0.92	0.94 ± 0.04	0.94	0.92	0.92	0.92	0.93	0.92	0.92	0.92	0.96	0.98	0.98
SVM-RF	0.88	0.92 ± 0.03	0.91	0.87	0.88	0.86	0.86	0.97	0.96	0.96	1.00	1.00	1.00
RF	0.88	0.93 ± 0.03	0.93	0.87	0.88	0.86	0.86	0.97	0.94	0.94	1.00	0.99	0.99
DT	0.87	0.87 ± 0.04	0.86	0.85	0.85	0.86	0.86	0.86	0.85	0.85	0.99	0.94	0.94
MLP-RF	0.85	0.94 ± 0.03	0.90	0.83	0.83	0.83	0.83	0.97	0.96	0.96	1.00	0.99	0.99
LightGBM	0.85	0.90 ± 0.03	0.91	0.81	0.89	0.74	0.93	0.87	0.91	0.88	0.99	0.96	0.96
Bagging	0.83	0.90 ± 0.04	0.89	0.78	0.89	0.70	0.93	0.91	0.89	0.85	1.00	0.97	0.97
MLP-DT	0.81	0.86 ± 0.05	0.86	0.78	0.79	0.77	0.77	0.98	0.96	0.96	1.00	0.96	0.96
CatBoost	0.81	0.82 ± 0.05	0.79	0.78	0.78	0.78	0.83	0.88	0.85	0.8	0.92	0.86	0.85
KNN	0.81	0.95 ± 0.02	0.81	0.77	0.8	0.76	0.76	0.81	0.81	0.81	1.00	1.00	1.00
CNN-LSTM	0.77	0.81 ± 0.05	0.78	0.75	0.75	0.76	0.76	0.9	0.81	0.81	0.93	0.85	0.85
CNN-BiLSTM	0.75	0.82 ± 0.06	0.79	0.72	0.72	0.72	0.72	0.92	0.83	0.83	0.95	0.88	0.88
CNN-GRU	0.73	0.81 ± 0.05	0.77	0.70	0.70	0.70	0.70	0.89	0.79	0.79	0.93	0.85	0.85
BiLSTM	0.67	0.71 ± 0.07	0.68	0.63	0.64	0.63	0.63	0.83	0.72	0.72	0.83	0.74	0.74
CNN	0.67	0.77 ± 0.04	0.67	0.62	0.63	0.62	0.62	0.83	0.72	0.71	0.89	0.80	0.8
AdaBoost	0.64	0.71 ± 0.06	0.69	0.51	0.63	0.43	0.80	0.74	0.64	0.42	0.85	0.79	0.75
GRU	0.63	0.73 ± 0.06	0.65	0.56	0.58	0.56	0.56	0.80	0.7	0.69	0.82	0.74	0.73
MLP	0.63	0.72 ± 0.07	0.63	0.56	0.58	0.56	0.56	0.82	0.64	0.62	0.77	0.67	0.66
LSTM	0.62	0.74 ± 0.05	0.65	0.56	0.57	0.56	0.56	0.81	0.70	0.70	0.83	0.75	0.74
SVM	0.52	0.64 ± 0.06	0.55	0.49	0.49	0.49	0.49	0.7	0.66	0.66	0.71	0.68	0.67
NB	0.46	0.52 ± 0.07	0.53	0.46	0.54	0.54	0.54	0.64	0.49	0.48	0.67	0.55	0.54

**Note:** The models include traditional ML algorithms (KNN: k-Nearest Neighbors; NB: Naive Bayes; SVM: Support Vector Machine; DT: Decision Tree; RF: Random Forest), ensemble learning algorithms (XGBoost: Extreme Gradient Boosting; LightGBM: light gradient boosting machine; CatBoost: Categorical Boosting; AdaBoost: Adaptive Boosting; Bagging: Bootstrap Aggregating), hybrid models (SVM-RF: Support Vector Machine-Random Forest; MLP-RF: Multi-Layer Perceptron-Random Forest; MLP-DT: Multi-Layer Perceptron-Decision Tree), and deep learning algorithms (MLP: Multi-Layer Perceptron; CNN: Convolutional Neural Network; LSTM: Long Short-Term Memory; GRU: Gated Recurrent Unit; BiLSTM: Bidirectional Long Short-Term Memory; CNN-LSTM: Convolutional Neural Network-Long Short-Term Memory; CNN-BiLSTM: Convolutional Neural Network-Bidirectional Long Short-Term Memory; CNN-GRU: Convolutional Neural Network-Gated Recurrent Unit).

### 3.3 Optimal model analysis

**Fig. 5:**
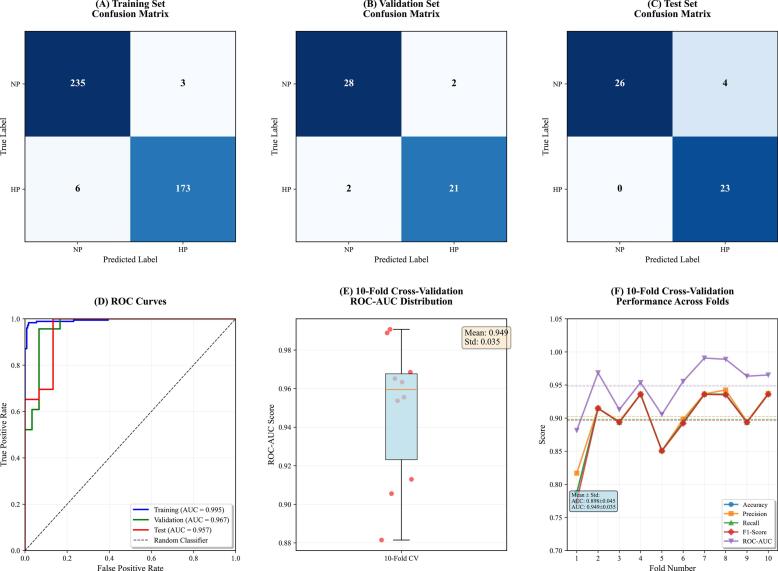
Performance Evaluation of the XGBoost Model. (A-C) Confusion matrices for training, validation, and test sets showing classification results for GZ and SF samples. (D) ROC curves demonstrating excellent discriminative ability (AUC: Training = 0.995, Validation = 0.967, Test = 0.927). (E) Boxplot of ROC-AUC scores from 10-fold cross-validation showing model stability (Mean = 0.949 ± 0.035). (F) Consistency of key metrics across 10-fold cross-validation. Results confirm high accuracy and robust generalization capability.

The XGBoost model showed very good performance in the discrimination of feeding patterns of yak milk with accuracies of 97.8%, 92.5%, and 92.5% on the training, validation, and test sets, respectively (Figs. 5A-C). Its good discriminatory power was confirmed by ROC analysis as well, which produced AUC values of 0.995, 0.967, and 0.927 for the same datasets (Fig. 5D). Model stability was confirmed using 10-fold stratified cross-validation, with an average AUC of 0.949 ± 0.035, consistent among all 10 folds (Figs. 5E-F). Together, these metrics show that the XGBoost model can accurately and reliably differentiate between GZ and SF patterns in yak milk.

### 3.4 Partial dependence and ICE analysis

**Fig. 6:**
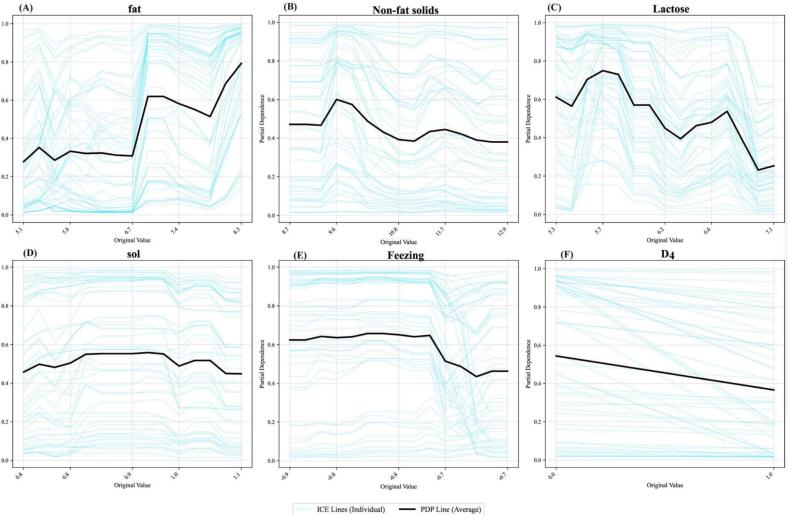
ICE and Partial Dependence Analysis. ICE plots (blue lines) and PDP (black lines) showing feature effects on model predictions for six variables: fat, non-fat solids, lactose, sol, freezing, and D_4_. ICE lines represent individual conditional expectations, while PDP lines show average marginal effects. The x-axis shows original feature values and y-axis shows partial dependence (0.0–1.0). Fat and lactose demonstrate positive associations with model output, while D_4_ shows negative correlation. (For interpretation of the references to colour in this figure legend, the reader is referred to the web version of this article.)

ICE and PDP analyses showed the marginal effects of key features (Fig. 6). Fat content showed the strongest positive effect, and the largest slope was found at 6.7–7%, a finding that identifies it as a critical determinant of the GZ mode. Lactose showed a weaker positive correlation with PDP values increasing from 0.35 to 0.65 in the 4.5–8.5% range. ICE curves showed heterogeneity at individual levels at higher concentrations for both features, indicating possible interaction effects. Additionally, the D_4_ with lower model prediction output when D_4_ = 1 (November) compared to D_4_ = 0 (not November), indicating temporal influence of SF. Collectively, these results provide quantitative evidence that fat, lactose and feeding stage are the most discriminating characteristics in yak milk.

### 3.5 SHAP analysis

#### 3.5.1 Feature importance Bar chart analysis

**Fig. 7:**
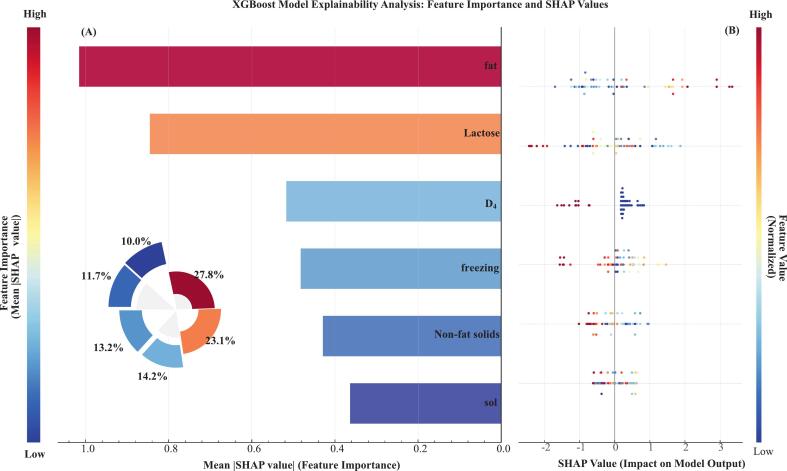
XGBoost model feature importance and influence analysis based on SHAP values (A) Global feature importance ranking. Bar lengths and pie chart percentages represent mean absolute SHAP values of each feature. (B) SHAP summary plot. Each point represents a sample, colors indicate feature value levels (red for high values, blue for low values), and horizontal position represents the SHAP value contribution of that feature to model prediction. (For interpretation of the references to colour in this figure legend, the reader is referred to the web version of this article.)

SHAP value analysis helped to understand the importance of features and decision-making process of the XGBoost model. Based on the average absolute SHAP values, the top four features were fat (27.8%), lactose (23.1%), D_4_ (14.2%) and non-fat solids (13.2%), and together explained 78.3% of the model's predictive power (Fig. 7A).

The SHAP summary plot (Fig. 7B) showed clear patterns of influence of each feature on predictions. High fat value resulted in positive SHAP values, which strongly indicated the GZ mode, while high lactose and high D_4_ values resulted in negative SHAP values, favoring the SF discrimination. Notably, fat and lactose showed largely inverse effects, indicating that there may be antagonistic effects that are reflective of underlying biological mechanisms, such as energy allocation and metabolic substrate competition in yak mammary glands.

#### 3.5.2 Waterfall plot individual explanation

**Fig. 8:**
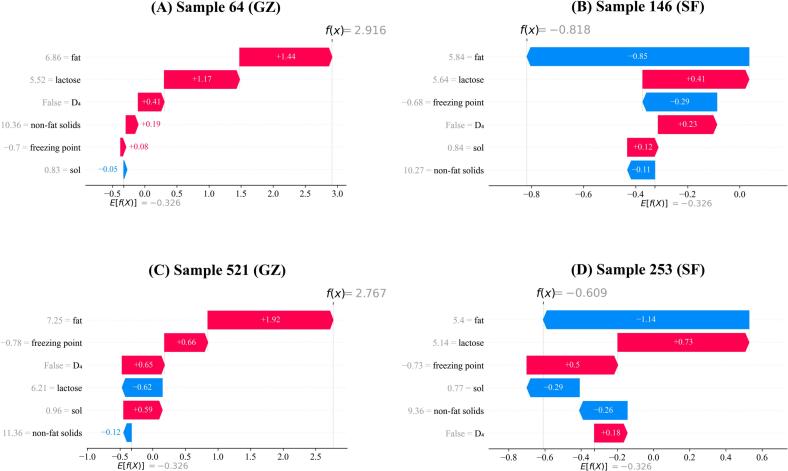
SHAP waterfall plot analysis for individual sample predictions. The x-axis represents SHAP values (log-odds), and the y-axis shows features with their corresponding values. Red bars indicate positive contributions toward GZ classification, blue bars indicate negative contributions, and bar length represents contribution magnitude. E[f(X)] denotes the baseline expected value, while f(x) represents the final prediction output. (For interpretation of the references to colour in this figure legend, the reader is referred to the web version of this article.)

SHAP waterfall plot analysis of two randomly selected samples of each feeding group revealed different discrimination mechanisms (Fig. 8). Predictions for GZ samples were driven mainly by fat content as we can see from sample 64 (fat contribution +1.44) and sample 521 (fat contribution +1.92) that were enough to override the other negative features. In contrast, SF sample classification seemed to rely on the combination of multiple features. For example, sample 146 was classified by the combined influences of fat (−0.85), freezing point (−0.29) and D_4_ (+0.23) with sample 253 displaying a similar trend. These results suggest that fat is a dominant feature in GZ identification, while proper SF discrimination involves the assessment of several interacting features.

#### 3.5.3 Dependency plot scatter analysis

**Fig. 9:**
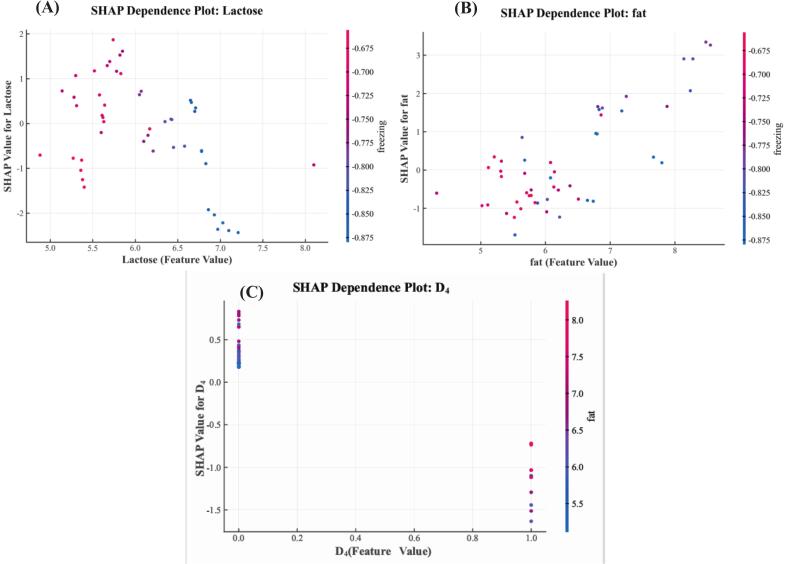
SHAP dependency plots for key features. The plots show how a feature's value (x-axis) affects the model's prediction (y-axis SHAP value). Each point represents one sample. The colour of each point shows the value of a second feature, which helps visualize their interaction. The subplots show the effects of: (A) Lactose, colored by freezing Point; (B) Fat, colored by freezing Point; and (C) SF stage (D_4_), colored by fat.

SHAP dependency plots (Fig. 9) revealed nonlinear interaction effects features. Lactose showed threshold behavior with high levels of lactose (> 6.0%) favoring SF mode and the combination of “high lactose + low freezing point” further improving SF predictions (Fig. 9A). The fat - freezing point interaction showed interval-dependent patterns as low fat levels (< 6.0%), high freezing point strengthened SF discrimination while high fat levels (> 6.5%), low freezing point promoted GZ discrimination (Fig. 9B). The D_4_-fat interaction showed that D_4_ = 1 and low fat content was an important discriminating signal for SF mode (Fig. 9C). These patterns of interactions are evidence of the model's high-precision discrimination by capturing complex, nonlinear combinations of features.

#### 3.5.4 Decision path and attribution pattern clustering

**Fig. 10:**
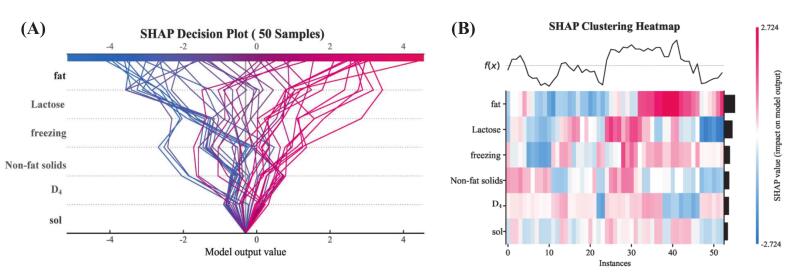
SHAP decision plots and clustering heatmap. (A) SHAP decision plots for 50 representative samples. Each line represents a sample's prediction path, starting from the baseline value (E[*f(x)*]) at the bottom, accumulating each feature contribution, and finally reaching the model output value *f(x)* at the top. Line colors represent final prediction value magnitude. (B) Hierarchical clustering heatmap based on SHAP values. The x-axis shows sample instances ordered by attribution similarity, and the y-axis shows features. Heatmap colors represent SHAP value magnitude (red for positive, blue for negative), intuitively displaying attribution patterns defining different sample clusters. The curve above the heatmap shows corresponding sample model raw output values *f(x)*. (For interpretation of the references to colour in this figure legend, the reader is referred to the web version of this article.)

SHAP decision plots and clustering analysis (Fig. 10) showed heterogeneity in model decision-making. The clustering heatmap (Fig. 10B) revealed two different attribution patterns: samples 5–24 (first cluster) were characterized by negative SHAP contributions from fat, corresponding to SF predictions *(f(x)* < 0), and samples 26–55 (second cluster) were characterized by positive SHAP contributions from fat, corresponding to GZ predictions *(f(x)* > 0). Samples within the intermediate range (23–26) had *f(x)* values that changed from negative to positive and crossed the classification decision boundary with the fat SHAP values changing accordingly. This transitional pattern may reflect individual variation among yaks, possibly affected by factors such as genetic background or health status, although these relationships need to be investigated further to confirm them.

## 4 Discussion

### 4.1 XGBoost algorithm performance analysis

The superior performance of the XGBoost model can be attributed to its robustness in capturing complex nonlinear relationships and high-order interactions among milk components (Burnwal & Jaiswal, 2023). This finding is in line with the recent dairy research where XGBoost has been shown to perform better than linear regression and RF in tasks from predicting proteins (Durgun, 2024) to large-scale classification of raw milk (Chung et al., 2022) and detection of adulteration (Chu et al., 2024). While RF also worked reasonably well, the sequential boosting mechanism of XGBoost was found to be more effective than the parallel bagging mechanism in identifying subtle compositional differences between feeding regimes (Ferreira & Figueiredo, 2012; Syam & Kaul, 2021).

In contrast, traditional algorithms did not perform well because of structural limitations compared to the specific characteristics of the yak milk data. The low accuracy of NB (46%) indicates its inability to capture strong biological correlations between components such as the positive association between fat and protein (Wang, Zhou, et al., 2023). Similarly, SVM (52%) had a hard time to deal with the high-dimensional time-dependent feature space in this study (Kirk et al., 2022; Verleysen & François, 2005). Deep learning models also demonstrated poor performance, mainly due to the small sample size (*n* = 523) which is insufficient to support the large number of parameters needed for neural network convergence (Cheng et al., 2025; Munkhdalai et al., 2023).

### 4.2 Biological interpretation of key discriminating features

SHAP interpretability analysis validated the biological plausibility of the decision-making process of the XGBoost model. Fat content as a key feature (relative importance 27.8%) aligns with basic dairy science (Bodkowski et al., 2024; Ding et al., 2013). Comparative studies have shown that milk fat content in GZ systems (4.2–4.5%) is significantly higher than in total mixed ration systems (3.8–4.0%, *p* < 0.001) (O'Callaghan et al., 2016). Long-term monitoring data from Pennsylvania State University also showed that pure grass-fed dairy cows had milk fat rates of 3.81%, while after SF with concentrates, milk fat contents decreased to 3.31% (Muler & Delahoy, 2003). These differences have been attributed to enhanced fiber fermentation under GZ conditions, which may promote acetic acid production and subsequently enhances milk fat synthesis (Grummer, 1991). Lactose, which accounted for 23.1% of model predictions, was the second most important feature. Under GZ conditions, the reduced protein content of pasture likely limits gluconeogenesis, possibly through activation of alternative metabolic pathways (García-Roche et al., 2021) or inhibition of key enzyme expression (Wang et al., 2022), thereby constraining lactose synthesis. Taken together, these findings offer a sound physiological basis for the importance of fat and lactose in the classification model.

SHAP dependence plots revealed fat-freezing-point interaction effects with physiological significance. In the low-fat range (< 6.0%) higher freezing points tended to move model predictions toward the SF mode. This is in accordance with the knowledge that the freezing point of yak milk is mainly controlled by the osmotic pressure of water-soluble components and is negatively correlated with fat content (Bouisfi & Chaoui, 2018; Otwinowska-Mindur et al., 2017). HP diets in SF systems boost milk yield (Craig et al., 2022), but mammary solid synthesis rates are relatively constant, resulting in a dilution effect. Peterson and Freeman (1966) also reported that freezing points in GZ herds (−0.539 °C) were lower than freezing points in mixed-diet herds. Furthermore, GZ conditions increase the proportion of unsaturated fatty acids and decrease saturated fatty acids, which affects the physical properties of milk fat (Elgersma, 2015). These patterns suggest that the model may capture the composite effects of feeding regimes on milk physicochemical properties.

The negative contribution of D_4_, a temporal variable, may reflect the combined effects of seasonal pasture decline and prolonged SF exposure. D_4_ corresponds to the autumn-winter transition when pasture becomes withered and nutritional value decreases, which in turn influences the composition of GZ yak milk. Li et al. (2011) reported that the milk fat content of cattle in cold seasons was 35.7% higher than in warm seasons, whereas Elemanova et al. (2024) found that the milk fat content of yaks reached its peak in spring and decreased throughout the summer. The D_4_-fat interaction implies that the model captures regulatory mechanisms that integrate the influences of time, season and feeding regime.

### 4.3 Feature interaction effects and multidimensional interpretability analysis

This study created a comprehensive validation framework by combining ICE/PDP and SHAP methodologies. ICE analysis found that fat had the highest individual heterogeneity around 7% concentration; notably, high fat concentrations (> 6.5%) coupled with low freezing points resulted in high positive SHAP values, suggesting that fat effects are modulated by freezing point differences. SHAP dependency plots of the interactions between lactose and freezing point also accounted for the divergence in lactose ICE curves at high concentrations (> 7.0%). The consistency of the results between these complementary methods adds to the robustness of the interpretation, showing that the XGBoost model offers both high predictive accuracy and reliable interpretability, while reducing the biases of single-method approaches.

### 4.4 Limitations and future research

This study achieved strong technical results but still has limitations. First, there was a limitation of sample representativeness, as the data set contained only 523 samples from two experimental sites in Qinghai Province. Variations in altitude, climate and grassland types in other regions of the Qinghai-Tibet Plateau may constrain the generalizability of the model. Second, the study was mainly based on routine milk composition data and did not include detailed biochemical indicators such as the fatty acid profile or milk protein fractions, and did not quantify environmental factors such as temperature and humidity. Future studies should try to increase sampling in various ecological regions across the Qinghai-Tibet Plateau, increase sample size, and build more complete sets of features, including detailed biochemical and environmental parameters, to increase model accuracy and enhance its applicability.

## 5 Conclusions

This study evaluated 21 ML algorithms on 523 samples of yak milk and found that XGBoost was the best model with an accuracy of 92% and an AUC of 0.94. Interpretability analysis showed that fat content (27.8%) and lactose (23.1%) were the main features of the model predictions. The model was able to capture complex interactions between features such as fat, lactose and freezing point, which are consistent with known physiological principles. This framework enables cost-effective authentication of feeding patterns based on routine compositional data providing practical support for yak milk quality control and certification. However, the model's regional applicability requires further validation, and future work should include additional biochemical and environmental features.

## CRediT authorship contribution statement

**Bo Hu:** Writing – original draft, Visualization, Software, Investigation, Formal analysis, Data curation, Conceptualization. **Lu Sun:** Investigation, Data curation. **Haiyue Wu:** Resources, Investigation, Data curation. **Rong Hu:** Investigation, Data curation. **Zhongxin Yan:** Writing – review & editing, Supervision, Project administration, Funding acquisition, Conceptualization.

## Declaration of competing interest

The authors declare that they have no known competing financial interests or personal relationships that could have appeared to influence the work reported in this paper.

## Data Availability

All data and materials relevant to this research article will be made available by the corresponding author, Dr. Zhongxin Yan (yzx990019@163.com), upon reasonable request.

## Appendix A Supplementary data

Supplementary material 1 Image 1

Supplementary material 2 Image 2
