# Supplementary figures and images for: Interpretable machine learning for yak milk feeding pattern discrimination: Integrating XGBoost with multidimensional explainability analysis
Source: Food Chem X. 2026 Jan 14;33:103541. doi: 10.1016/j.fochx.2026.103541 (PMC12853049; doi:10.1016/j.fochx.2026.103541)

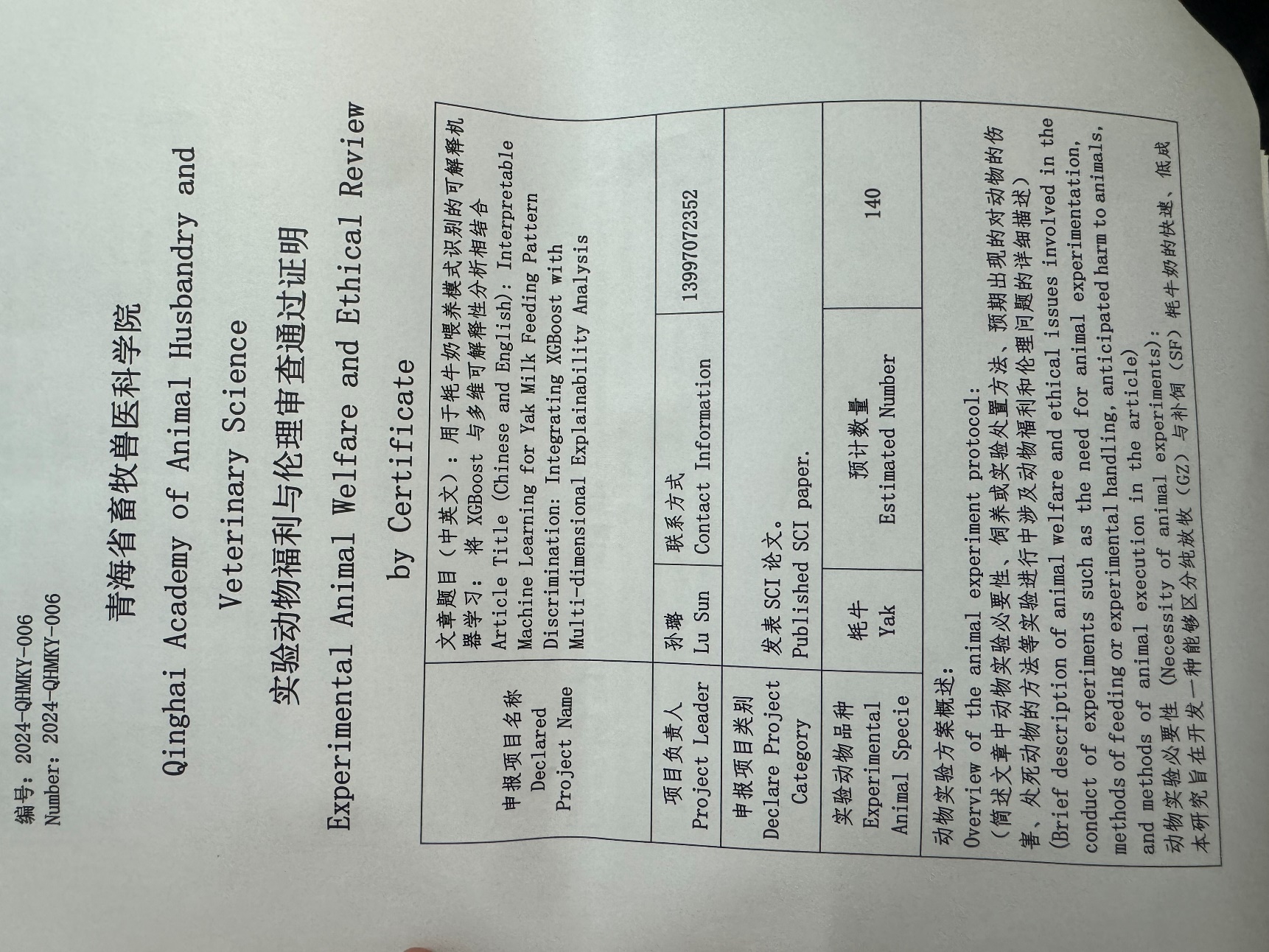


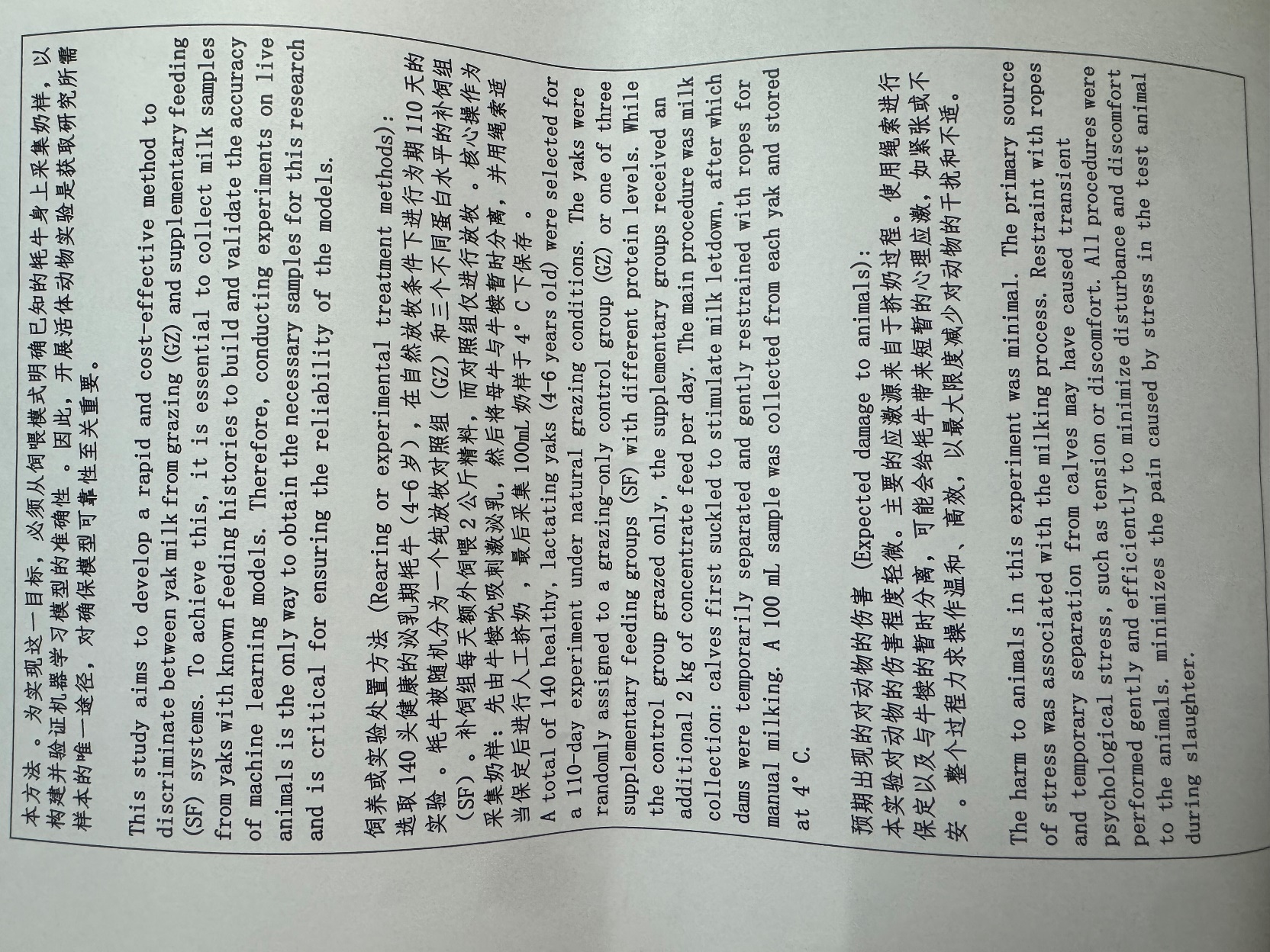


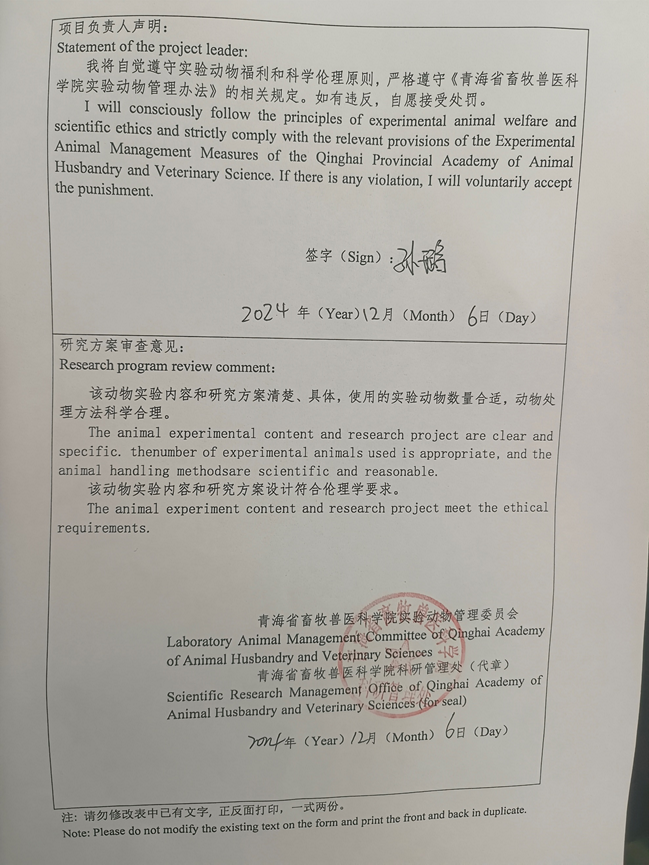

Supplement: Supplementary file 2 — Supplementary material 2 [file mmc2.docx]
